# Triamcinolone Acetonide-Assisted Visualization and Removal of Vitreous Cortex Remnants in Retinal Detachment: A Prospective Cohort Study

**DOI:** 10.3390/diagnostics15151854

**Published:** 2025-07-23

**Authors:** Francesco Faraldi, Carlo Alessandro Lavia, Daniela Bacherini, Clara Rizzo, Maria Cristina Savastano, Marco Nassisi, Mariantonia Ferrara, Mario R Romano, Stanislao Rizzo

**Affiliations:** 1 Ophthalmology Unit, Surgical Department, A.O. Ordine Mauriziano,10128 Turin, Italy; faraldi@retinatorino.it; 2 Department of Neurosciences, Psychology, Drug Research, and Child Health, Eye Clinic, Azienda Ospedaliero Universitaria Careggi, University of Florence, 50134 Florence, Italy; daniela.bacherini@gmail.com (D.B.); clararizzo2@gmail.com (C.R.); 3 Ophthalmology Unit, Fondazione Policlinico Universitario A.Gemelli IRCCS, 00136 Rome, Italy; mariacristina.savastano@gmail.com (M.C.S.); stanislao.rizzo@gmail.com (S.R.); 4 Department of Head and Neck Medicine, Università Cattolica del Sacro Cuore, 00168 Rome, Italy; 5 Department of Clinical Sciences and Community Health, University of Milan, 20122 Milan, Italy; m.nassisi@gmail.com; 6 Fondazione IRCCS Ca’ Granda Ospedale Maggiore Policlinico, 20122 Milan, Italy; 7 Eye Clinic, Department of Medical and Surgical Specialties, Radiological Sciences and Public Health, University of Brescia, 25123 Brescia, Italy; mariantonia.ferrara@gmail.com; 8 Department of Biomedical Sciences, Humanitas University, 20072 Pieve Emanuele-Milan, Italy; mario.romano.md@gmail.com; 9 Department of Ophthalmology, Humanitas Gavazzeni-Castelli, 24125 Bergamo, Italy; 10 Italian National Research Council, CNR, 56124 Pisa, Italy

**Keywords:** proliferative vitreoretinopathy, rhegmatogenous retinal detachment, vitreous cortex remnants, retinal toxicity, triamcinolone acetonide

## Abstract

**Background/Objectives**: In rhegmatogenous retinal detachment (RRD), vitreous cortex remnants (VCRs) may contribute to the development and progression of proliferative vitreoretinopathy (PVR). This study aimed to evaluate potential toxicity and trauma secondary to VCRs visualization and removal during pars plana vitrectomy (PPV) for RRD. **Methods**: Prospective study on patients with primary RRD who underwent PPV. Imaging assessment included widefield OCT (WF-OCT), ultra-WF retinography and fundus autofluorescence (FAF). During PPV, a filtered and diluted triamcinolone acetonide (TA) solution (20 mg/mL) was used to evaluate the presence and extension of VCRs, removed using an extendible diamond-dusted sweeper (EDDS). After six months, retinal and retinal pigment epithelium toxicity and retinal trauma due to VCRs removal were investigated. **Results**: Retinal reattachment was achieved in 21/21 cases included in the study. No signs of retinal or RPE toxicity were detected and WF-OCT performed in the areas of VCRs removal revealed an intact inner retinal architecture in the majority of eyes, with minor and localized inner retinal indentations in 4 cases. **Conclusions**: VCRs visualization and removal using TA and EDDS appears to be safe, with no retinal toxicity and very limited and circumscribed mechanical trauma. This approach may contribute to reducing the risk of postoperative PVR.

## 1. Introduction

Proliferative vitreoretinopathy (PVR) stands as the main cause of surgical failure, following primary surgery for rhegmatogenous retinal detachment (RRD) [1,2,3]. Although it remains a subject of debate, on the basis of several studies and surgical experiences, the presence of vitreous cortex remnants (VCRs) is believed to play a significant role in the onset and progression of PVR [4]. Consequently, several authors advocate for the visualization and accurate removal of VCR as key maneuvers to reduce the risk of PVR and, thereby, the recurrence of retinal detachment [5,6,7].

Vitreous cortex remnants represent the outermost lamellae of the posterior vitreous cortex remaining adherent on the retinal surface following an anomalous posterior vitreous detachment (PVD) associated with vitreoschisis [8,9]. Based on their anatomical location, VCRs are typically classified into macular VCRs (m-VCR) and peripheral VCRs (p-VCR) [9].

Owing to their inherent transparency, intraoperative visualization of VCR under standard endoillumination is challenging and is typically facilitated through the intravitreal injection of triamcinolone acetonide (TA) [10]. TA crystals are able to adsorb on residual vitreous collagen fibrils, leading to their visualization of VCR as sheet-like or patchy areas exhibiting a whitish, granular appearance attributable to the presence of the adherent TA particles [11,12]. Despite being effective and widely used in vitreoretinal surgery, the potential cytotoxic effect of TA and its preservative, benzyl alcohol (BA), on neurosensory retinal cells and retinal pigment epithelium (RPE) cells, has been documented, in particular in vitro [13,14].

The removal of VCR can be performed using various surgical instruments. Traditional techniques have employed tools such as end-gripping forceps, silicone-tipped needles and retinal brushes, the latter mostly used in the past. More recently, the adoption of disposable loops or membrane scrapers has become increasingly prevalent, particularly when used in conjunction with beveled vitrectomy probes [15,16,17,18]. These instruments aim to achieve a more controlled and selective peeling of residual vitreous cortex while minimizing trauma to the underlying retina.

Besides efficacy, frequent criticisms on the feasibility and need of routine p-VCR removal are raised based on technical concerns, including the significant time required to achieve an adequate VCR removal, potential retinal toxicity associated with TA, and the risk of iatrogenic retinal injury [19,20]. This study aims to evaluate the potential structural damage to outer and inner retina associated with TA-assisted removal of VCR using an extendible diamond-dusted sweeper (EDDS) during pars plana vitrectomy (PPV) in patients affected by primary RRD. Particular attention is given to the potential trauma induced by the use of the EDDS, a relatively recent tool, for the removal of VCRs.

## 2. Materials and Methods

### 2.1. Study Design

This is a prospective observational study on patients diagnosed with primary uncomplicated RRD who underwent PPV with p-VCRs removal between January and December 2024. The study was approved by the Local Ethics Committee (protocol number 155/2020, general registry number n°11486, Inter-Hospital Ethics Committee, San Luigi Gonzaga Hospital, Orbassano, Italy) and adhered to the tenets of the Declaration of Helsinki. Informed consent was obtained from all patients.

### 2.2. Patients Selection

Consecutive patients were enrolled according to the following inclusion criteria:

- age ≥ 18 years
- diagnosis of primary uncomplicated RRD
- RRD treated by PPV +/− scleral buckling
- intraoperative evidence of peripheral VCR
- minimum follow-up of 6 months

Exclusion criteria were previous PPV; RD secondary to other ocular diseases (e.g., vitreoretinal dystrophies, inflammatory eye disease, etc.); pre-existing significant ocular comorbidities such as diabetic retinopathy, advanced glaucoma, inherited vitreoretinal dystrophies; PVR ≥ grade C at presentation [21]; impossibility to acquire good quality imaging.

### 2.3. Preoperative Assessment

Preoperative evaluation was performed by an experienced vitreoretinal surgeon (FF).

During the preoperative visit, the surgical approach was planned: PPV alone or combined with scleral buckling. A combined procedure with cataract surgery was planned and performed when deemed necessary.

All patients underwent comprehensive preoperative assessments, which included measurement of best-corrected visual acuity (BCVA) using a Snellen chart, applanation tonometry, slit-lamp biomicroscopy and dilated fundus examination. Additionally, each index eye underwent an imaging protocol including optical coherence tomography (OCT) and ultrawidefield (UWF) imaging.

OCT was performed using the Heidelberg Spectralis HRA + OCT device (Heidelberg Eye Explorer, Version 1.11.2.0; software V7.0.1; Heidelberg Engineering, Heidelberg, Germany). The protocol consisted of a sequence of 97 Horizontal B-scans covering an area of 55°, recorded in high resolution mode (1536 A-scans per B-scan), with an interscan distance of approximately 120 μm. The “automatic real-time” function was set at 16. OCT combined with infrared (OCT + IR) images were captured, centered on the macula and in quadrants where acquisition was possible due to the presence of an RRD.

Ultrawidefield fundus color photography and fundus autofluorescence (FAF) imaging were performed using Optos Silverstone, Software v5.1.11.119967 (Optos—a Nikon Company—Dunfermline, Fife, UK), to assess the preoperative status of the retina and the vitreoretinal interface. One or more images were acquired to obtain a satisfying visualization of the retinal detachment and care was taken to minimize imaging adjacent lids and lashes.

Optical biometry was performed in all phakic patients.

### 2.4. Surgical Technique

In all cases, a standard three-port 25-gauge PPV was performed using the Constellation Vision System (Alcon Laboratories, Inc., Fort Worth, TX, USA), under peribulbar anesthesia. A fourth sclerotomy was created to insert a 25-G chandelier illumination system (Alcon Laboratories, Inc., Fort Worth, TX, USA). When necessary, phacoemulsification with IOL implantation in the capsular bag was performed. A three-piece IOL (Sensar AR40e, Johnson and Johnson Surgical Vision, Irvine, CA, USA) was preferred in this context due to its higher stability. If deemed necessary, a silicone encircling band (Stile #40, Labtician Ophthalmics, Oakville, Canada) was placed at 12.5 mm from the limbus and secured to the sclera with 5/0 polyester sutures, which were repositioned as needed during surgery. Following core vitrectomy, retinal tractions at the break(s) were relieved with the vitreous cutter. Then, a filtered (High flow filter, 20 micron; BVI Visitec, Waltham, MA, USA) and diluted TA solution (20 mg/mL, prepared from Kenacort 40 mg/mL; Bristol Myers Squibb, Rome, Italy) was injected for PVD assessment. Ref. [4] previously reported this TA formulation as effective for intraocular use during PPV, particularly for the detection of VCRs. Moreover, using a filtered and diluted TA preparation may facilitate multiple intraoperative applications, improve ease of removal, and reduce the risk of concentration-dependent TA-related toxicity [4]. If a complete PVD was not present, it was mechanically induced with the vitreous cutter. After PFCL injection up to the vascular arcades to stabilize the posterior pole and peripheral vitrectomy, TA was re-injected accurately towards each retinal quadrant to detect VCRs. A backflush soft tip was used to aspirate the TA particles sedimented on the retinal surface, revealing p-VCRs in the areas where the particles remained adherent. The location and extension of the area of VCR removal were recorded during the surgical procedure. Moreover, all surgical procedures were recorded and stored in an external memory for postoperative evaluation. Vitreous cortex remnants were removed using a 25-G extendible diamond-dusted sweeper (EDDS; DORC, Zuidland, The Netherlands). After 360° peripheral vitreous gel shaving under scleral depression and complete fluid–air exchange, endolaser was applied where necessary. Finally, one of the following endotamponades was used: SF6 18% (Alchimia, Ponte San Nicolò, Italy), C3F8 18% (Alchimia, Italy) or silicone oil (SO) 1000 cSt (RS-OIL 1000 cS; Alchimia, Ponte San Nicolò, Italy).

### 2.5. Postoperative Examinations

A complete ophthalmic examination, identical to the baseline assessment, was performed at 1 day, 1 week, and 1, 3, and 6 months following surgery. In cases where SO was used and a retinal reattachment was confirmed at the 1-month follow-up, tamponade removal was scheduled within 3 months of the initial surgery, with subsequent follow-up visits arranged as described above.

Patients who underwent surgery with documented presence and removal of VCR were included in the study and recalled within 6 months postoperatively to assess potential toxicities of TA and trauma induced by VCR removal.

**Figure 1:**
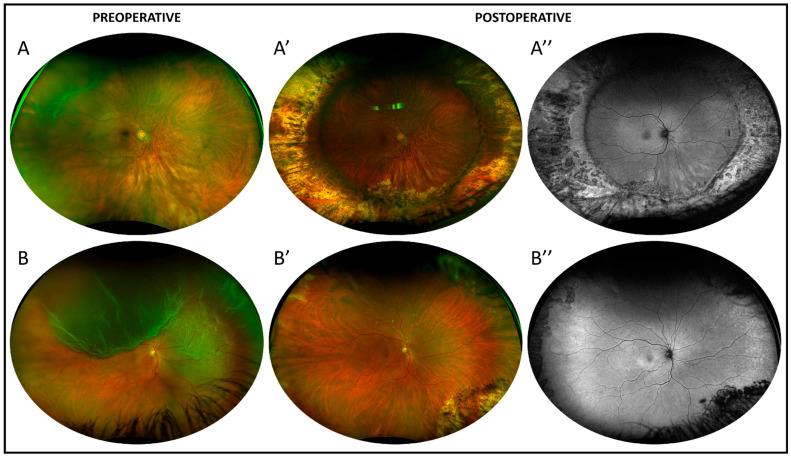
Preoperative and postoperative ultrawidefield (UWF) assessment. (**A**,**B**): Preoperative UWF retinography of two right eyes presenting a retinal detachment in the temporal quadrants (**A**) and in the superior and nasal quadrants (**B**). (**A′**,**B′**,**A″**,**B″**): postoperative UWF retinography (**A′**,**B′**) and autofluorescence (**A″**,**B″**). Patient A underwent cataract surgery + vitrectomy + scleral buckle and gas tamponade (C3F8). Patient B underwent vitrectomy with gas tamponade (C3F8). No signs of retinal toxicity were observed in the postoperative UWF assessment.

**Figure 2:**
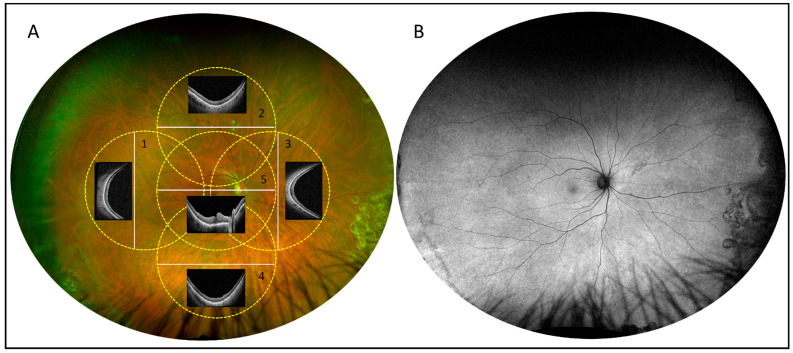
Postoperative OCT assessment of retinal toxicity. (**A**): Postoperative ultrawidefield retinography from a patient who underwent pars plana vitrectomy with gas tamponade. The five circles (yellow dotted lines) represent the ≈55° areas observed using wide field OCT to detect outer retina/retinal pigment epithelium (RPE) toxicity following surgery. The white lines represent the position of the B-scan as reported within each circle (lines 1–5). (**B**): Postoperative ultrawidefield fundus autofluorescence from the same patient. No outer retina/RPE toxicity was observed using the described imaging techniques.

Structural damage to the outer retina was evaluated through the standardized imaging protocol used at the preoperative assessment (Figure 1) and, as retinal reattachment was achieved in all cases, OCT + IR images centered on the macula and across the four quadrants (superior, temporal, inferior, nasal) were acquired, as shown in Figure 2.

**Figure 3:**
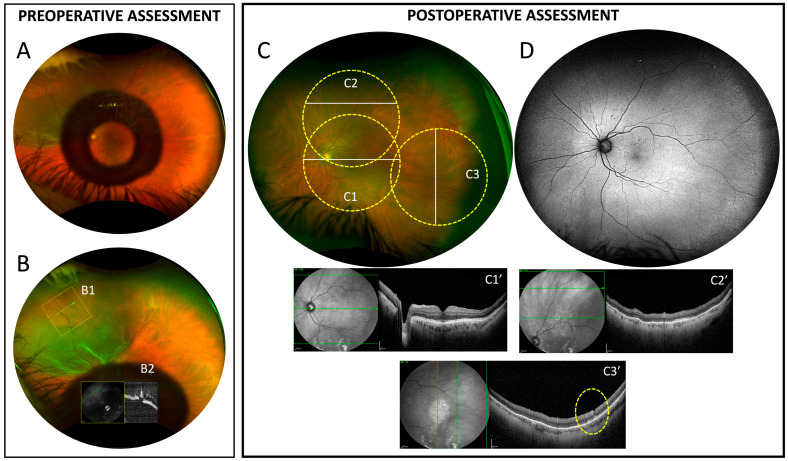
Preoperative and postoperative assessment. Postoperative evaluation of potential retinal trauma. (**A**,**B**): preoperative ultrawidefield retinography from a patient affected by primary rhegmatogenous retinal detachment involving the nasal and superior quadrants with a retinal break at XI hours (**B1**), imaged with a peripheral OCT scan (**B2**). The patient previously underwent a Kamra inlay implantation to correct presbyopia. The inlay was removed before vitreoretinal surgery. (**C**,**D**): postoperative ultrawidefield retinography and autofluorescence. The three dotted yellow circles (**C1**–**C3**) represent the ≈55° areas acquired using wide field OCT within the areas of VCRs removal. The white lines represent the position of the B-scan as reported in figures (**C1′**–**C3′**, **green arrows**). In (**C3′**), the yellow dotted line underlines a small V-shaped inner retinal interruption caused during VCRs removal.

In addition, possible iatrogenic retinal damage induced by p-VCR removal using EDDS was assessed by performing peripheral OCT scans in the areas where intraoperative VCR removal had been recorded, as shown in Figure 3.

## 3. Results

Of 62 patients diagnosed with primary RRD evaluated at our ophthalmology unit during the study period, 11 patients underwent scleral buckling and were therefore excluded from the study; of the remaining 51 patients, 42 underwent PPV alone and 9 PPV combined with encircling band. Among the 51 patients, p-VCR were identified in 21 cases (41.2%). The mean postoperative follow-up was 8.5 months. Baseline patient characteristics and surgical details are presented in Table 1.

Primary anatomical success, defined as complete retinal reattachment following gas reabsorption or SO removal, was achieved in all cases. Silicone oil was used in 7/21 patients (33%) and was removed at a mean of 88 ± 12 days after the primary surgery. Mean BCVA improved from 20/160 preoperatively to 20/63 at last follow-up; no cases of unexplained vision loss were recorded.

No signs for suspected TA-induced damage of the outer retina were detected on UWF retinography, fundus autofluorescence, and widefield OCT (Figure 1 and Figure 2). In particular, OCT imaging revealed neither alterations in the RPE (e.g., RPE defects, mottling, intraretinal migration) nor any irregularities or disruptions observed in the or outer retinal layers (e.g., interdigitation zone and/or ellipsoid and/or ELM irregularities or disruption). Additionally, UWF–FAF demonstrated an absence of hyper/hypo-autofluorescence irregularities at the posterior pole as well as throughout the retinal periphery (Figure 1).

Widefield OCT performed in areas of p-VCR removal showed an intact inner retina in 18/21 (86%) cases, while small indentations of the inner retina were observed in 3/21 (14%) cases. In particular, these areas appeared as small, V-shaped discontinuities of the inner retina, not associated with surrounding inner retinal thinning or other retinal tissue alterations (Figure 3).

## 4. Discussion

In this study, we investigated the safety of TA-assisted visualization and EDDS-assisted removal of p-VCRs in patients with primary RRD, with a postoperative imaging assessment focused on the evaluation of potential retinal toxicity and trauma related to these maneuvers.

The presence of both m-VCR and p-VCR is believed to be associated with an anomalous PVD [9,17,22]. Sebag and collaborators firstly described the anomalous PVD as determined by vitreous gel liquefaction in absence of concurrent complete dehiscence at the vitreoretinal interface [23,24]. The concomitant splitting of the posterior vitreous cortex, known as vitreoschisis, and the level at which this splitting occurs, is particularly important in the pathophysiology of proliferative vitreoretinopathies [25,26]. In particular, a relatively anterior splitting may leave a thicker and hypercellular membrane on the retina resulting in the development of a macular pucker; whereas, the thin and hypocellular membrane following a more posterior splitting may be responsible for the formation of macular holes exerting centrifugal tangential forces [27]. Within the peripheral retina, following anterior splitting of the vitreous cortex, the resulting p-VCR may act as a scaffold for fibrocellular proliferation and play an active role in extracellular matrix remodeling with hyalocites mediating its contraction, eventually leading to the development of PVR [28]. Consistently, histological studies on PVR membranes supported an association between VCR and PVR, as the latter was characterized by the presence of myofibroblasts and retinal pigment epithelial cells surrounded by vitreous collagen [29].

Based on the above-mentioned considerations, over the last years, several authors have proposed TA-driven VCR removal during RRD surgery as surgical maneuver to reduce the risk of PVR development and, thus, recurrent RD [9]. In recent years, several authors evaluated the possible impact of p-VCR on PVR development. Van Overdam et al. [17], in a retrospective analysis, reported p-VCRs in 56/159 eyes (35.2%) and performed VCRs removal in 37 of them (66%); after a minimum follow-up of 6 months, they observed retinal redetachment in 4 eyes (2.5%), attributed to peripheral VCR/PVR in 3 eyes (75%). Rizzo et al. [7], found VCRs in all 413 cases analyzed and removed them in 190 eyes (46%); after a minimum follow-up of six months, all the cases of retinal redetachment (21 eyes, 5.1%) were attributed to p-VCR/PVR. In our study, all 21 eyes underwent PPV with VCRs removal and no RD recurrences were observed.

Two main concerns related to TA-aided VCR removal are the potential structural damage to the outer retina and iatrogenic damage induced by the staining with TA and the removal maneuver, respectively.

The intraocular use of steroids dates back to the early 1970s, and since then, various molecules and formulations have been employed for the treatment of inflammatory retinal diseases, such as uveitis, macular edema secondary to vasculopathies, and vitreoretinopathies [30,31,32]. Among them, TA represented for several decades an effective molecule for the treatment of inflammatory retinal diseases as well as for intraoperative use during vitrectomy, for its anti-inflammatory properties and its capability to stain the vitreous [10,33]. Although off label, TA has been long and effectively used for the intraoperative staining of the vitreous [34]. However, some concerns have been raised regarding its safety based on the experimental evidence of potential retinal toxicity [35,36,37]. Interestingly, Szurman et al. reported that toxicity, even at very low doses, can be detected only in case of direct contact of TA crystals with RPE cell surface as the formation of TA crystals exceeds the solubility equilibrium [13,38]. Then, particular attention has been paid to the potential toxicity of BA, commonly used at 0.99% as TA-preservative, as it has been demonstrated that this molecule is able to induce immediate production of reactive oxygen species leading to RPE necrosis and mitochondrial apoptosis [14,39]. It is worth noting that the evidence regarding TA-induced toxicity in vivo and in vitro is controversial and there is a certain incongruence between laboratory and clinical data on TA toxicity, with and without BA [40]. This may be attributable to the presence of the inner limiting membrane (ILM), that represents a natural barrier for TA penetration into the retinal tissue, as well as of the choriocapillaris, that may contribute to clearance of TA from the outer retina, further reducing intra-retinal drug retention [13,37,38].

While different intraocular steroids have been proposed and are currently employed for the treatment of inflammatory and vascular retinal pathologies, TA still represents nowadays the preferred option for vitreous visualization and removal during vitreoretinal surgery [12,41]. Moreover, during vitreoretinal surgery, TA exposure to the retinal tissue is unremarkable as compared to that after intravitreal injection for medical purposes, furtherly reducing time-dependent potential retinal toxicity [42].

Several strategies can be employed to minimize the amount of TA injected, while preserving an adequate visualization of the VCRs using the smallest possible amount of TA. This was achieved by diluting TA, most often to 20 mg/mL, a maneuver that also facilitates its removal, by reducing the quantity of TA injected into the vitreous chamber or by employing combined physical and mechanical techniques. Dell’Omo et al. [18], in a study on 103 eyes affected by RRD, used 0.5 mL of TA at a concentration of 40 mg/mL together with a loop-based mechanical identification technique for the identification of VCRs. In our study, TA was filtered and diluted to reach a final concentration of 20 mg/mL and was administered an average of 2.7 times per surgical intervention. Using this protocol, no signs of retinal/RPE toxicity were found on WF and UWF OCT and FAF. This may be in line with a better safety profile of diluted TA formulations compared to undiluted ones as well as with the protective role of an intact ILM [36]. Finally, although a new lutein-based vitreous dye may represent a safer alternative to TA, its efficacy in staining the VCR may be limited and lower to that of TA.

In addition to the visualization and identification techniques for VCRs, which should aim for the highest possible efficacy and safety, there are also VCR removal techniques that present potential difficulties. Indeed, removal of p-VCR is a challenging and time-consuming procedure, posing a risk of inducing retinal damage that could be either related to the traction on the surrounding retinal tissue (which may lead to the formation of retinal tears) and to the direct retinal trauma induced by the maneuver itself [19]. In the past, VCRs removal has been performed using end-gripping forceps, silicone-tipped cannulas or the vitrectomy probe [43]. Subsequently, with the aim of simplifying and standardizing the procedure, dedicated instruments like nitinol loops and diamond duster scrapers have been proposed for a better and easier management of VCR [7,16,18]. In this study, extensive VCRs removal maneuvers were performed using the EDDS, with no intraoperative complications. Consistent to our findings, the effectiveness and ease to use of diamond duster scrapers for VCRs removal has been previously reported [7,44]. Various inner retinal defects have been described following peeling procedure and related to mechanical forces due to scraping, pulling and pushing on the retinal tissue, including retinal dimples, localized nerve fiber layer injury, partial thickness grooves in the ILM. Leung et al., in a series of patients who underwent PPV for macular pucker and macular hole with membrane peeling using a flexible silicone-tipped cannula covered with diamond fragments, observed the presence of inner retinal defects that on histopathology appeared as sharp, linear and partial thickness grooves in the ILM that were attributed to the DDMS use [45]. To the best of our knowledge, this is the first study assessing the potential iatrogenic trauma associated with EDDS- aided VCR removal using peripheral OCT in areas where intraoperative VCRs were documented and removed. Promisingly, OCT scans revealed only a limited number of cases (3/21), small and isolated disruptions of the inner retinal continuity, similar to DONFL on the en-face images, likely to be related to direct damage from VCR scraping. Although no comparative studies between diamond duster scrapers and ninitol loops for VCRs removal are available, ninitol loops have also been associated with postoperative alterations of the inner retinal following peeling maneuvers. In particular, Dell’Omo et al. [18], performing a 55° postoperative OCT centered on the macular region, observed dissociated optic nerve fiber layer (DONFL) at 3 and 6 months postoperatively in 19 out of 103 eyes following the use of a nitinol loop for VCR removal, attributing this finding to the concomitant, unintentional, peeling of the ILM. It should also be emphasized that different degrees of VCR adhesion can be observed during removal. Indeed, in some instances, the VCR can be removed as a whole with a maneuver akin to a hyaloidorexis, by initiating a linear incision outside the vascular arcades, elevating a continuous layer of VCR up to the posterior border of the vitreous base, and completing the removal with vitreous cutter aspiration. In other cases, stronger adhesion makes it difficult to elevate a VCR flap, possibly due to differences in the depth of the vitreoschisis or in the stage of the PVR process.

The limitations of this study include the small sample size and the use of a filtered, preserved TA formulation, which renders it subject to significant intra- and inter-operator variability [46]. The limited number of patients analyzed is attributable to the high number and lengthy duration of pre- and postoperative imaging examinations; as this is a pilot study investigating potential toxicity and trauma in particular within the peripheral retina, a huge number of WF-OCT scans were performed. Finally, it could be argued that we did not evaluate functional outcomes beyond BCVA. However, our investigations were specifically planned to detect the clinical signs typically associated with dye-induced retinal toxicity; in addition, in case of RRD, baseline conditions may substantially influence the results of functional testing, thereby limiting their interpretability in this context.

In conclusion, the use of TA for VCRs visualization combined with their mechanical removal using EDDS is a challenging, time-consuming but safe adjunct in PPV for RRD with no retinal toxicity, very limited and circumscribed mechanical trauma and the advantage of potentially reducing the risk of postoperative PVR. Further studies are warranted to refine these techniques, compare different instruments for VCRs removal, and validate their long-term clinical benefits.

## Figures and Tables

**Table 1:** Preoperative and intraoperative data from the 21 patients included in the study.

*Case n*	*RRD LOCATION and EXTENSION*	*MACULA ON/OFF*	*PRESENCE OF PVR (GRADE)*	*VCR AREA (QUADRANTS)*	*SURGERY*	*VCR REMOVAL (Q)*	*TAMPONADE*	*ENDOLASER*
***1***	S, 2 q	On	A	4	Phaco + PPV	4	SF6	360°
***2***	S, 1 q	On	No	1	PPV	1	SF6	Sectorial
***3***	S e N, 2 q	On	A	3	PPV	2	C3F8	360°
***4***	T and I, 3 q	Off	B	2	PPV + SB	2	SO	360°
***5***	Total, 4 q	Off	B	4	Phaco + PPV + SB	4	SO	360°
***6***	S and T, 3 q	Off	B	4	Phaco + PPV	4	C3F8	360°
***7***	T, 1 q	On	No	1	PPV	1	SF6	Sectorial
***8***	S, 2 q	On	No	1	PPV	1	SF6	Sectorial
***9***	Total, 4q	Off	A	3	Phaco + PPV	3	SO	360°
***10***	S and T, 3 q	Off	A	2	PPV	2	SF6	360°
***11***	N and I, 2 q	On	No	2	Phaco + PPV	2	C3F8	360°
***12***	Total, 4q	Off	B	3	PPV + SB	2	C3F8	360°
***13***	S, 2 q	On	No	1	Phaco + PPV	1	SF6	Sectorial
***14***	N and I, 3q	On	A	2	PPV	2	C3F8	360°
***15***	I, 1 q	Off	B	1	Phaco + PPV	1	SO	Sectorial
***16***	I, 2 q	Off	B	3	PPV + SB	2	SO	360°
***17***	Total, 4q	Off	B	4	PPV + SB	3	SO	360°
***18***	T and I, 2q	On	No	2	PPV	2	C3F8	360°
***19***	I, 2 q	On	A	2	PPV	2	SO	Sectorial
***20***	S and T, 3 q	Off	No	3	Phaco + PPV	3	SF6	360°
***21***	Total, 4q	Off	B	3	PPV + SB	3	C3F8	360°

RRD: rhegmatogenous retinal detachment; location and extension are expressed as number of quadrants (Q) involved; S: superior, N: nasal, T: temporal, I: inferior; PVR: proliferative vitreoretinopathy; VCR: vitreous cortex remnants; Phaco: phacoemulsification + intraocular lens implantation, PPV: pars plana vitrectomy, SB: scleral buckling.

## Data Availability

The original contributions presented in this study are included in the article. Further inquiries can be directed to the corresponding author.

## Abbreviations

The following abbreviations are used in this manuscript:

RRD	Rhegmatogenous retinal detachment
VCRs	vitreous cortex remnants
PVR	proliferative vitreoretinopathy
TA	triamcinolone acetonide
PPV	pars plana vitrectomy
OCT	optical coherence tomography
WF-OCT	widefield OCT
FAF	fundus autofluorescence
EDDS	extendible diamond-dusted sweeper
RPE	retinal pigment epithelium
PVD	posterior vitreous detachment
m-VCRs	macular VCRs
p-VCRs	peripheral VCRs
UWF	ultrawidefield
BCVA	best-corrected visual acuity
SO	silicone oil
BA	benzyl alcohol
ILM	inner limiting membrane
DONFL	dissociated optic nerve fiber layer
